# Biotechnological Potential of *Yucca decipiens* Trel Based on Proximate Composition, Multi-Elemental Analysis, and Nursery Growth Performance

**DOI:** 10.3390/biotech15020026

**Published:** 2026-03-25

**Authors:** Selena del Rocío Martínez-Betancourt, Jorge Cadena-Iñiguez, Laura Araceli López-Martínez, Janet María León Morales, Ramón Marcos Soto-Hernández, Gerardo Loera-Alvarado, Víctor Manuel Ruiz-Vera, Concepción López-Padilla

**Affiliations:** 1 Programa de Innovación en Manejo de Recursos Naturales, Colegio de Postgraduados, Iturbide 73, Salinas de Hidalgo 78600, San Luis Potosi, Mexico; selenitabetancourt@gmail.com (S.d.R.M.-B.); gerardo.loera@colpos.mx (G.L.-A.); vmanuel@colpos.mx (V.M.R.-V.); 2 Coordinación Académica Región Altiplano Oeste, Universidad Autónoma de San Luis Potosí, Carretera Salinas-Santo Domingo 200, Salinas de Hidalgo 78600, San Luis Potosi, Mexico; araceli.lopez@uaslp.mx (L.A.L.-M.); janet.leon@uaslp.mx (J.M.L.M.); concepcion.lopez.p@gmail.com (C.L.-P.); 3 Programa de Botánica, Colegio de Postgraduados, Campus Montecillo, km. 36.5 Carretera México-Texcoco, Montecillo, Texcoco 56264, State of Mexico, Mexico; msoto@colpos.mx

**Keywords:** *Yucca decipiens*, biomass valorization, ICP-MS, proximate composition, mineral profile, free amino acids, semi-arid plants, sustainable biomass production, nutraceutical applications

## Abstract

*Yucca decipiens* is a native species from arid and semi-arid regions with emerging nutritional and biotechnological potential. This study evaluated its proximate composition, elemental profile determined by inductively coupled plasma mass spectrometry (ICP-MS), and growth performance under nursery conditions. Proximate analysis revealed a high dietary fiber content in leaves (58.93%) and higher carbohydrate levels in stems (28.83%). Free amino acid content was significantly higher in stems (2.75 g histidine equivalents kg^−1^) than in leaves (1.76 g kg^−1^). Multi-elemental profiling (63 elements) showed organ-specific accumulation patterns, with essential macro- and micronutrients predominantly concentrated in leaves, including potassium (28,334 ppm) and calcium (15,345 ppm), while iron was the most abundant trace element in stems (1253 ppm). Principal component analysis (PCA) revealed clear organ-specific mineral partitioning between leaves and stems, indicating differentiated physiological roles and potential selective biomass utilization. Growth assessment conducted over a two-year period demonstrated steady biomass accumulation and good adaptive performance under nursery conditions. Overall, the results highlight the emerging nutritional and agroindustrial relevance of *Yucca decipiens* for applications in semi-arid environments.

## 1. Introduction

The semi-arid ecosystems of Mexico cover approximately 71 million hectares and are characterized by high agroclimatic vulnerability. In these regions, rural communities rely heavily on non-timber forest resources, particularly endemic plant species, which play a central role in subsistence strategies and local economies [1,2,3]. These resources support traditional medicine, food systems, and handicrafts and represent an important source of income for rural populations [4]. The genus *Yucca* L. comprises approximately 40 to 50 perennial, shrubby, and arboreal species, of which nearly 30 are endemic to Mexico, accounting for more than 50% of the global diversity of the genus [5,6]. Species of this genus have been traditionally used in food, cosmetic, and medicinal applications due to their content of secondary metabolites such as saponins and polyphenols, which confer recognized biological and therapeutic properties [7,8,9]. Despite increasing scientific interest, several *Yucca* species remain poorly studied, particularly with respect to the chemical composition of their leaves and stems. In this context, *Y. decipiens*, an endemic species of the Mexican semi-arid, remains poorly characterized in terms of its proximate and mineral composition, which currently limits its agro-industrial and biotechnological valorization [10]. Assessing its potential as a source of plant biomass and bioactive metabolites may support sustainable use strategies and contribute to the socio-economic development of rural communities [11]. Similar approaches have been applied to underutilized species from the Fabaceae family, such as *Canavalia ensiformis* and *Phaseolus lunatus*. These studies have demonstrated that non-conventional plant resources can provide proteins and compounds with functional properties of agro-industrial relevance [12]. Beyond their nutritional and industrial value, perennial plant species adapted to semi-arid environments, such as *Y. decipiens*, may also play a role in environmental applications, particularly phytoremediation.

This strategy exploits the ability of plants to remove, immobilize, or transform contaminants, including heavy metals, from soils or water [13]. Certain species classified as hyperaccumulators can tolerate and sequester high concentrations of metals in their tissues without severe physiological damage, reaching concentrations of hundreds or thousands of parts per million, depending on the element and species involved [13,14,15]. Therefore, evaluating the mineral composition of *Y. decipiens* may provide insights into its capacity to accumulate or tolerate specific elements, supporting its potential use in phytoremediation and sustainable land management strategies in semi-arid regions.

Although the present study does not address the structural identification or biological evaluation of individual compounds, it focuses on the proximate and mineral characterization of leaves and stems as a preliminary step toward biomass quality assessment. The research was conducted in accordance with the principles of the Convention on Biological Diversity and the Nagoya Protocol, which promote sustainable use of biodiversity, equitable benefit-sharing, and respect for traditional knowledge [16].

In this context, expanding scientific knowledge on underutilized native plant species is essential to foster biotechnological applications with tangible social and environmental impact [17]. Considering that *Yucca decipiens* exhibits slow growth and that water resources in semi-arid environments are limited, large-scale nursery production has been proposed as an alternative cultivation strategy. Harvesting plants at approximately 30 months may allow the production of stem and leaf biomass suitable for flour production with potential commercial value.

## 2. Materials and Methods

### 2.1. Plant Material and Sample Preparation

Leaves and stems of *Yucca decipiens* Trel. were obtained from mass nursery production of 30-month-old plants located at 22.625956 N, −101.710719 W (San Luis Potosí, Mexico). Samples were randomly collected from independent plants to ensure representativeness.

Plant material was washed and disinfected using a 5% NaOCl solution, cut into small fragments, and dried in a forced-air oven (FELISA^®^ TE-HV30D, Guadalajara, Jalisco, Mexico) at 35 °C until constant weight (seven days). The dried material was then ground and sieved (ASTM standard stainless-steel sieves) to obtain a particle size fraction between 0.150 and 0.212 mm and stored in airtight containers until analysis. All reagents were of analytical grade (Sigma-Aldrich^®^, St. Louis, MO, USA; J.T. Baker^®^, Tultitlán, Mexico). Each determination was performed in triplicate using independently prepared sub-samples.

### 2.2. Proximate Analysis

Moisture content was determined according to AOAC method 966.02 [18]. Five grams of powdered sample were dried at 110 °C for 3 h. Moisture percentage was calculated using Equation (1):

Moisture (%) = (B − A)/(B − C) × 100(1)

where A is the weight of the empty capsule, B is the weight of the capsule with fresh sample, and C is the weight of the capsule with dried sample.

Crude protein was determined using the Micro-Kjeldahl method [18], calculating total nitrogen and using a nitrogen-to-protein conversion factor of 6.25.

Total carbohydrates were quantified according to AOAC 974.06 [19], and total sugars were determined following NMX-F-312-NORMEX-2016 (2016). Lipid content was measured using the Soxhlet extraction method [20], and dietary fiber was determined using the Weende method 962.09/90 [20].

Ash content was determined following AOAC method 942.05/90 [20]. Ash percentage was calculated using Equation (2):

Ash (%) = D/C × 100(2)

where C is the weight of the dry sample and D is the weight of the calcined residue.

All proximate values were expressed on a dry weight basis (% DW).

### 2.3. Mineral Analysis

Mineral content was determined after open acid digestion following a modified protocol [21]. Briefly, 0.5 g of dried and ground sample was digested with 10 mL of ultrapure HNO_3_ and allowed to stand for 12 h at room temperature. The samples were then subjected to controlled heating until approximately 1 mL of digest remained. Subsequently, 10 mL of concentrated H_2_O_2_ were added dropwise to complete mineralization. The digests were diluted to 25 mL using Class A volumetric flasks. Indium (In) and iridium (Ir) were used as internal standards at a concentration of 25 ng mL^−1^.

Elemental determination was performed using Inductively Coupled Plasma Mass Spectrometry (ICP-MS) (iCAP™ RQ, Thermo Fisher Scientific^®^, Bremen, Germany) operating in kinetic energy discrimination (KED) mode to minimize spectral interferences. A total of 63 elements were analyzed. For elements with multiple naturally occurring isotopes, a representative isotope was selected for quantification to avoid redundancy. For example, calcium was quantified using ^44^Ca in KED mode, lead using ^208^Pb, and uranium using ^238^U.

Calibration was carried out using multi-element standard solutions, and quality control included procedural blanks and certified reference materials. The limits of detection (LOD) for the analyzed elements were in the low ng mL^−1^ range under the applied analytical conditions.

### 2.4. Determination of Free Amino Acids

Free amino acids were quantified using the Cd–ninhydrin colorimetric method [22,23,24]. The reagent consisted of 0.8 g of ninhydrin dissolved in 80 mL of ethanol (99.5%), 10 mL of 0.5 M glacial acetic acid, and 1 g of CdCl_2_ dissolved in 1 mL of distilled water.

For the analysis, 0.2 mL of extract was mixed with 1.5 mL of reagent, heated at 84 °C for 5 min, and then cooled in an ice bath. Absorbance was measured at 505 nm. Quantification was performed using an L-histidine standard calibration curve ranging from 0 to 0.6 mM (0–93.09 µg mL^−1^).

### 2.5. Growth Assessment

The growth performance of *Y. decipiens* was evaluated under uniform nursery conditions using a completely randomized sampling design. Ten representative plants were randomly selected and monitored over a two-year period. Growth measurements were recorded at six-month intervals. This exploratory assessment was conducted under controlled nursery conditions, considering the slow growth rate of the species and the logistical constraints associated with long-term longitudinal monitoring of perennial plants.

### 2.6. Statistical Analysis

Data were analyzed using R (programming language) (v4.1.1) within RStudio (v2021.09.0). Results are expressed as mean ± standard deviation of three independent determinations.

Comparisons between leaves and stems were performed using Student’s *t*-test at a significance level of *p* < 0.05.

Principal Component Analysis (PCA) was conducted using standardized mineral concentration data to evaluate multivariate patterns and discrimination between plant tissues. Elemental concentrations obtained by ICP-MS were used as variables in the multivariate analysis. When elements presented multiple isotopes, a single representative isotope was selected to avoid redundancy in the PCA dataset.

### 2.7. Data Availability

The datasets generated and analyzed during the current study are available from the corresponding author upon reasonable request. No large-scale public database deposition was required, as the study did not involve genomic, transcriptomic, or proteomic datasets.

### 2.8. Ethical Considerations

This study did not involve humans or animals and therefore did not require ethical approval. Plant material was obtained from nursery production systems and handled in accordance with institutional and national regulations. The research was conducted under the principles of sustainable use of biodiversity and in accordance with the Convention on Biological Diversity and the Nagoya Protocol.

### 2.9. Use of Generative Artificial Intelligence

Generative artificial intelligence tools were used solely to assist with language editing and improvement of clarity in the manuscript. No AI tools were used for data generation, data analysis, figure production, or interpretation of results.

## 3. Results

### 3.1. Proximate Analysis

The proximate composition of *Y. decipiens* leaves and stems is presented in Table 1. Dietary fiber was the predominant component in both organs, reflecting a strong structural biomass contribution. Significant differences (*p* < 0.05) were observed between leaves and stems for carbohydrates, sugars, protein, ash, and fiber content.

Stems exhibited higher total carbohydrate and sugar levels, whereas leaves showed a significantly greater fiber proportion. Ash content was also higher in stems, indicating differential mineral accumulation, while moisture content did not differ significantly between tissues. These results confirm organ-specific differences in the distribution of major biomass components between leaves and stems.

### 3.2. Free Amino Acid Content

The total free amino acid content, expressed as histidine equivalents due to the analytical standard used, was significantly higher (*p* < 0.05) in stems (2.75 ± 0.07 g histidine equivalents kg^−1^) than in leaves (1.76 ± 0.02 g histidine equivalents kg^−1^).

### 3.3. Mineral Analysis

The mineral composition of *Y. decipiens* leaves and stems is presented in Table 2. Significant differences (*p* < 0.05) were observed between organs for several elements, indicating differential mineral allocation within the plant and suggesting organ-specific mineral partitioning that may influence biomass quality and potential functional applications.

### 3.4. Trace Element Analysis

The concentration of essential trace elements in Yucca decipiens leaves and stems is presented in Table 3. Statistically significant differences were detected between organs (*p* < 0.05), indicating tissue-specific accumulation patterns. Iron (Fe) was the most abundant trace element in stems, reaching 1253.39 ppm, suggesting a greater capacity for micronutrient storage in this organ.

### 3.5. Toxic Trace Element Composition in Y. decipiens Organs

Analyses of toxic trace elements in *Y. decipiens* revealed statistically significant differences (*p* < 0.05) between leaves and stems. Aluminum (Al) and silicon (Si) were among the most abundant non-essential trace elements detected in both organs. Aluminum showed similar concentrations in both organs, whereas silicon predominated in leaves (402.05 ± 0.01 ppm) (Table 4).

### 3.6. Distribution of Toxic and Rare-Earth Trace Elements in Y. decipiens

Various toxic trace elements were detected in the leaves and stems of *Y. decipiens*, including detectable concentrations of strontium, titanium, bismuth, and tin, as well as the presence of uranium (U), thorium, and thallium (*p* < 0.05; Table 5). In addition, rare-earth elements such as cerium, lanthanum, neodymium, and gadolinium were predominantly found in leaves. Overall, concentrations were higher in leaves than in stems, which may be associated with differences in mineral accumulation and translocation between organs.

### 3.7. Organ-Specific Metal Accumulation

Lanthanum (La) showed homogeneous concentrations in both organs (12.03 ppm in leaves and 12.04 ppm in stems). Dysprosium (Dy) was significantly higher in leaves (0.04 ppm) than in stems (0.02 ppm). Tungsten (W) and gold (Au) showed higher concentrations in stems (0.35 ± 0.01 ppm and 0.15 ± 0.01 ppm, respectively) (Table 6).

### 3.8. Multivariate Analysis (PCA)

**Figure 1:**
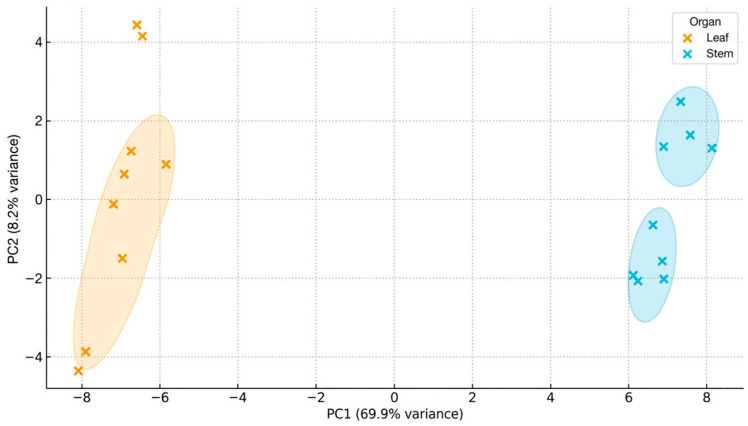
PCA scores of mineral composition in *Y. decipiens* leaves and stems.

Principal component analysis (PCA) was performed using the standardized concentrations of all quantified mineral elements in leaves and stems of *Y. decipiens*. The first principal component (PC1) explained 69.9% of the total variance, whereas PC2 accounted for an additional 8.2%, so that the first two components together captured 78.0% of the multivariate dispersion (Figure 1).

PC1 clearly separated leaves (negative scores) from stems (positive scores), indicating a strong organ-dependent mineral partitioning pattern. This axis was mainly associated with macrominerals such as K, Ca, Mg, Na and P, which showed the highest loadings in absolute value, together with several micro and trace elements.

PC2, which explained 8.2% of the variance, was primarily related to elements such as Si and selected trace metals, refining within organ differences but without overlapping leaf and stem clusters.

Silicon showed one of the highest loadings in PC2, suggesting that this element contributed to secondary mineral differentiation between organs. Silicon has been associated with structural reinforcement, improved drought tolerance, and enhanced resistance to abiotic stress in plants growing in arid and semiarid environments. Therefore, the relative contribution of Si to PC2 may reflect adaptive physiological mechanisms that support the structural stability of *Y. decipiens* under xeric conditions.

Table 7 and Table 8 show cumulative explanatory variance and statistical loadings of each principal component for the mineral composition of *Y. decipiens* leaves and stems. Leaves consistently exhibited higher concentrations of essential macro- and micronutrients (K, Ca, Mg, P, Zn, Cu, Mn), while stems showed relatively higher scores associated with certain trace elements.

### 3.9. Growth Dynamics of Y. decipiens Under Nursery Conditions

Growth dynamics of *Y. decipiens* were evaluated under nursery conditions over a 30-month period. Plants exhibited gradual increases in leaf number, plant height, and aboveground biomass throughout the evaluation period. Leaf production followed a progressive pattern across sampling intervals, reflecting steady vegetative development under controlled nursery conditions.

Leaf growth in *Y. decipiens* during the evaluation period showed a slow and progressive pattern, consistent with the literature on this species. At the beginning of the experiment (day zero), the plants showed no foliar development. After 124 days, the leaves reached an average height of 19–20 cm, showing a relatively low initial increase, characteristic of slow-growing species.

After 340 days of nursery cultivation, the most notable increase in leaf height was observed, with average values between 36 and 42 cm, indicating a phase of sustained growth. This increase continued until 519 days, with heights between 42 and 49 cm, and subsequently until 703 days, where 49–56 cm was recorded. Finally, at the end of the evaluated period (884 days), the leaves showed a stabilization in height, with constant values of approximately 55 cm.

**Figure 2:**
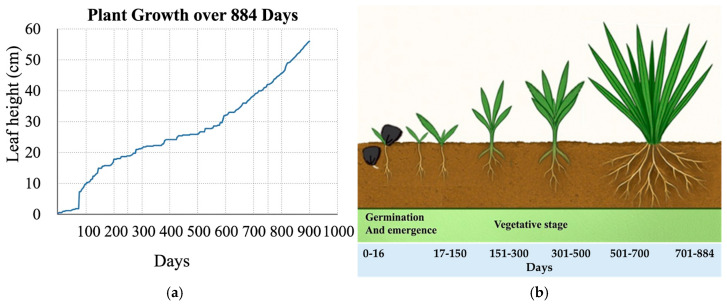
(**a**) *Yucca decipiens* Trel plant in growth phase at 30 months. (**b**) Growth of leaves and stems of *Y. decipiens* Trel. in nursery over 30 months.

These results confirm that *Y. decipiens* is a slow-growing species that can be managed through nursery cultivation. Under this approach, it is possible to achieve quality foliar biomass in an approximate period of 30 months, providing a feasible method for mass production as a source of flour and metabolites for the agroindustry (Figure 2a,b).

## 4. Discussion

According to the data in Table 1, the high fiber content in the leaves of *Y. decipiens* (58.93 ± 2.22%) was significantly higher (*p* < 0.05) than that of the stem (40.10 ± 1.78%). Compared to the value reported for the mesocarp of the *Yucca mixtecana* fruit (51.65 ± 2.05%) [25], this suggests its potential as a source of functional fiber. By comparison, the flowers of *Yucca filifera* and *Y. treculeana* have considerably lower levels of crude fiber (6.91–15.67%) [26].

Although crude fiber represents only a fraction of total dietary fiber, these data demonstrate the presence of relevant structural components in different organs of the genus *Yucca*. From an agronomic perspective, this fiber-rich profile supports the potential use of leaves as a targeted biomass fraction under managed propagation systems, allowing selective harvesting depending on the intended application.

Regarding total carbohydrates, the stem showed a significantly higher value (*p* < 0.05; 28.83 ± 1.37%) than the leaf (12.83 ± 0.57%). Total carbohydrates include not only simple sugars (monosaccharides and disaccharides) but also complex polysaccharides such as starches and fiber, while total sugars specifically refer to soluble sugars that provide readily available energy.

Total sugars were also significantly higher (*p* < 0.05) in the stem (23.37 ± 0.73%) compared to the leaf (9.63 ± 0.58%), indicating a greater accumulation of available carbohydrates. This organ-specific carbohydrate distribution suggests differentiated metabolic roles and supports organ-based valorization strategies for biomass utilization.

Regarding protein, the stem of *Y. decipiens* (10.88 ± 0.48%) showed a significantly higher content (*p* < 0.05) than the leaf (9.17 ± 0.40%). These levels are comparable to those reported for flowers of *Y. aloifolia* (12.5%) [27] and for flowers of *Y. treculeana* (19.12%), *Y. filifera* (18.42%), and *Agave mapisaga* Trel. (11.00%) [26].

Fat content was low in both organs, although slightly higher in the leaf (2.33 ± 0.46%) than in the stem (1.11 ± 0.68%). This difference was statistically significant (*p* < 0.05) and could be attributed to the presence of lipid compounds in the leaf cuticle that perform structural and protective functions.

Moisture levels in *Y. decipiens* were low and showed no significant differences (*p* > 0.05) between organs, with values of 6.09 ± 1.03% in the leaf and 5.78 ± 0.85% in the stem, corresponding to dry matter contents of 93.91 ± 1.03% and 94.22 ± 0.85%, respectively.

The higher concentration of free amino acids detected in stems suggests differences in nitrogen allocation between plant organs. When compared with other plant-derived materials, the amino acid content of *Y. decipiens* is lower than that reported for wheat flour (4.005 g kg^−1^) and buckwheat flour (3.461 g kg^−1^), as well as mixtures of both flours whose total amino acid contents range from 3.567 to 3.819 g kg^−1^ [28]. When expressed on a protein basis, values between 0.82 and 1.86 g 100 g^−1^ protein have been reported in other plant materials [29]. Differences in expression units among studies should be considered when making direct quantitative comparisons. Although the amino acid concentration in *Y. decipiens* is lower than that of conventional protein sources such as wheat, the presence of these compounds in leaves and stems highlights the nutritional potential of this species and supports its possible application as a functional plant biomass resource.

The ash content was slightly higher in the stem (7.52 ± 0.19%) than in the leaf (6.73 ± 0.11%) (*p* < 0.05), suggesting that the stem may concentrate a higher proportion of total minerals. In comparison, *Y. treculeana* petals have shown up to 13% of the ash content, with variations depending on the processing method [26]. The specific types of minerals present are described in the following section.

These compositional attributes highlight the potential of *Yucca decipiens* biomass as a raw material for the development of functional ingredients, mineral-enriched products, and other value-added biotechnological applications. To further understand the nutritional and technological value of this biomass, the multielemental composition was analyzed using ICP-MS.

The mineral composition observed in *Y. decipiens* reveals notable concentrations of several macroelements. Compared with other species of the genus such as *Y. filifera* and *Y. gloriosa*, *Y. decipiens* exhibited relatively higher levels of certain macroelements, particularly potassium (K) and calcium (Ca), which may reflect physiological adaptations to semi-arid environments. Unless otherwise stated, the elemental concentrations discussed in this section correspond to plant dry biomass and should not be directly interpreted as dietary intake levels.

The results presented in Table 2 show a differential distribution of minerals among the organs of *Y. decipiens*. Leaves exhibited higher concentrations of calcium, potassium, magnesium, and phosphorus, whereas stems were mainly associated with structural support and transport functions within the plant.

These findings are consistent with reports for *Capsicum* fruits, where mineral distribution is influenced by genotype, maturity stage, and environmental growing conditions [30]. Calcium (Ca) concentrations were significantly higher in the leaf (15,345.54 ppm) compared to the stem (7628.34 ppm), reflecting its structural role in the cell wall and its limited mobility through the phloem. Thus, the leaf contains approximately twice as much Ca as the stem, which is consistent with the well-known low phloem mobility of calcium and its structural function in plant tissues [31]. These values are higher than those reported in *Capsicum* sp. fruits, which range from 4.76–36.9 ppm (dry weight basis) [30]. In comparison, the epicarp of *Y. mixtecana* fruit has a Ca content of 0.24 ppm [25], while *Y. filifera* inflorescences showed values between 6.783 and 7.305 ppm [32], considerably lower than those obtained in this study for the leaf and stem of *Y. decipiens*.

Potassium (K) was the most abundant mineral in both plant organs, with values of 28,334.00 ppm in leaves and 20,190.31 ppm in stems. Its high concentration is associated with its role in osmoregulation and enzyme activation, as potassium is known to regulate cellular osmotic balance and activate numerous enzymes involved in plant metabolism [31]. Lower concentrations, ranging from 20,758 to 31,297 ppm, have been reported in *Y. filifera* inflorescences [32], while the epicarp of the *Y. mixtecana* fruit reached 150 ppm [25]. In comparison, in quelites such as *Amaranthus* spp., potassium (K) varied between 4600 and 9200 ppm [33], and in other species such as purslane (*Portulaca oleracea*), romerito (*Suaeda* spp.), or *huauzontle* (*Chenopodium berlandieri*), between 2790 and 18,623 ppm [9]. Even in edible ornamental flowers such as rose (*Rosa* sp.) and marigold (*Tagetes erecta*), concentrations were notably lower (1967.3 and 3808.7 ppm) [32]. These results highlight the high potential of *Y. decipiens* as a natural source of potassium.

Magnesium (Mg) showed an uneven distribution among the analyzed organs, with a notably higher concentration in leaves (8155.24 ppm) compared to stems (2299.34 ppm), which could be attributed to its role as a central component of chlorophyll. Much lower concentrations have been reported in *Y. filifera* flowers (3010 and 3021 ppm) [32], and in *Y. gloriosa*, values as low as 25.1 ppm [34].

In contrast, the epicarp of *Y. mixtecana* reached a significantly high concentration of 980 ppm, the highest reported for the genus [25]. Other authors have reported concentrations of approximately 2000 ppm in *Yucca* spp. flowers [35], reinforcing the evidence of interspecific and inter-organ variability, attributed to genetic, functional, and environmental factors. Sodium (Na), present in smaller quantities, showed similar values in both organs (252.81 ppm in leaves and 304.68 ppm in stems), suggesting a low role in primary physiological functions. In comparison, considerably lower concentrations (10 ppm) have been reported in *Y. mixtecana* [25]. In *Capsicum* sp. fruits, reported concentrations ranged from 97 to 452 ppm, depending on the genotype and environment [30].

Phosphorus (P), a vital element in nucleic acid synthesis and energy transfer, was more concentrated in the leaf (4845.25 ppm) than in the stem (3299.19 ppm). Reports for *Y. filifera* indicate high concentrations of 3548 ppm [32], and in Mexican quelites (*Amaranthus* spp.) values between 1645 and 12,810 ppm have been recorded [36]. These differences are explained by the intense metabolic activity of the leaves, which are the photosynthetically active tissues and require higher levels of phosphorus for processes such as photosynthesis and cellular energy production.

The high concentrations of essential macrominerals in foliar tissues indicate that leaves represent a nutritionally dense biomass fraction, whereas stems function primarily as structural and transport organs. This organ-specific mineral partitioning is particularly relevant for controlled production systems, as it supports targeted harvesting strategies aimed at maximizing nutritional value while minimizing potential toxicological risk associated with differential metal accumulation.

This mineral-rich profile also suggests potential biotechnological applications. Plant biomass enriched in essential elements such as potassium, calcium, and magnesium may be explored as a source of mineral-enriched functional ingredients, plant-derived supplements, or raw material for nutraceutical and agroindustrial formulations. In this context, the organ-specific mineral distribution observed in *Yucca decipiens* could support selective biomass processing strategies aimed at maximizing its nutritional and technological value.

Table 3 shows significant organ-specific accumulation of micronutrients and trace elements in *Y. decipiens* (*p* < 0.05) between leaves and stems. In general, Fe showed higher accumulation in stems, whereas Zn, Cu, and Mn were more concentrated in leaves, while Co showed greater accumulation in stems and Cr in leaves, reflecting species-specific physiological and edaphoclimatic influences.

This distribution pattern suggests differential roles in transport and storage, particularly for Fe in structural tissues, possibly related to sap conduction and redox processes linked to photosynthesis [37]. Fe values of around 58 ppm have been reported in *Yucca* spp. flowers [3], while in *Y. filifera* inflorescences, values ranged from 24.10 to 56 ppm [32]. Up to 10 ppm was recorded in the epicarp of *Y. mixtecana* fruit [25], and values in Mexican quelites leaves (*Amaranthus* spp.) varied between 33 and 93 ppm [9].

The leaves of *Y. decipiens* showed significantly higher concentrations (*p* < 0.05) of zinc (764.47 ppm) than the stems (703.65 ppm), which is consistent with its function as an enzyme cofactor and regulator of plant growth [38]. In *Yucca* spp. flowers, Zn values of 80 to 110 ppm were reported [35], while *Y. filifera* registered 4681 ppm [32] and *Y. gloriosa* only 25.4 ppm, demonstrating the high nutritional potential of *Y. decipiens* as a source of Zn. In Mexican quelites, the values ranged from 23.2 to 106 ppm [9].

Copper (Cu) and manganese (Mn) also accumulated primarily in leaves (256.71 ppm and 53.90 ppm, respectively), exceeding stem values (160.51 ppm and 23.82 ppm). Cu levels were higher than those reported in flowers of *Yucca* spp. (14 ppm) [35] and *Ravenia spectabilis* (21.73 ppm) [39]. Although the concentrations do not exceed permissible limits, due to the potential risk of toxicity from excessive accumulation, it is recommended to assess their safety before considering nutraceutical applications [40].

Mn showed higher values than those observed in *R. spectabilis* (39.1 ppm) [39], possibly reflecting a physiological adaptation of Yucca decipiens to arid soils rich in Mn. By comparison, cobalt (Co) was more abundant in stems (0.41 ppm), whereas chromium (Cr) showed higher concentrations in leaves (25.41 ± 3.19 ppm) compared to stems (21.95 ± 2.22 ppm). Molybdenum (Mo) and selenium (Se) were detected at low concentrations in both organs. Co levels remained within safe ranges.

Silicon (Si) is an element present in significant quantities in the inflorescences of *Yucca filifera*, with reported concentrations ranging from 55 to 243.8 ppm [32]. Although some forms of silicon can be toxic at high doses, its consumption in food is considered safe and has been linked to benefits for bone health, connective tissue integrity, and collagen metabolism [41]. The recommended daily intake for adults is approximately 30 mg day^−1^, so the consumption of these edible flowers in rural areas of Mexico could represent an alternative dietary source of silicon, with concentrations that do not exceed established limits.

Considering reference values, the estimated human intake varies between 5 and 40 µg day^−1^, while tolerable limits in soils are between 0.2 and 0.5 ppm, although some species grow in much higher concentrations (4000–10,000 ppm) [40]. Direct comparisons between plant concentration (ppm) and daily intake limits should be interpreted cautiously. Nevertheless, these reference values help contextualize the detected concentrations, which did not suggest an immediate toxicological risk.

Co is an essential micronutrient involved in fatty acid oxidation and DNA synthesis; iron deficiency causes anorexia, anemia, and bone fragility in ruminants [42,43]. However, an excess could disrupt iron homeostasis, so evaluating its bioavailability is recommended [44].

Taken together, these results indicate that *Y. decipiens* exhibits significant organ-specific accumulation of essential minerals such as Mn, Fe, Zn, Cu, and Co (*p* < 0.05). This distribution pattern may be influenced by species-specific physiological mechanisms as well as edaphoclimatic conditions [39]. The observed mineral partitioning highlights the ecological adaptability of the species and suggests potential for further investigation regarding its response to metal-enriched soils.

Due to its Fe and Zn content, *Y. decipiens* may represent a potential natural source of micronutrients; however, the bioavailability of these nutrients could be limited by antinutritional compounds present in the plant, such as phytates and tannins [45]. These findings highlight the species’ potential as a nutritional resource and underscore the importance of evaluating its safe and efficient use in human or animal feed.

The variations observed between leaves and stems of *Y. decipiens* could be related to edaphic or environmental factors, as has been described for lemon juice (*Citrus* sp.) from different regions of Argentina, whose elemental composition allowed its geographical classification [46].

These findings allow the evaluation of both the nutritional potential and the capacity of the species to bioaccumulate metals, suggesting possible applications in environmental monitoring or phytoremediation research in contaminated soils, as well as the need for further studies on food safety and bioavailability. The capacity of *Y. decipiens* to accumulate both essential and non-essential elements may also have relevance for environmental biotechnologies, including phytoremediation and biomonitoring strategies in metal-impacted soils.

Cadmium (Cd) showed significant differences (*p* < 0.05) between organs, with a higher concentration in the stem (0.28 ± 0.01 ppm) compared to the leaves (0.19 ± 0.01 ppm), which could indicate a preference for accumulating in structural tissues. These concentrations do not exceed the established safety limits for medicinal herbs (0.30 mg kg^−1^) [41]. Although comparisons with drinking water standards (0.003 μg L^−1^) [47] and weekly intake limits (490 μg day^−1^) [48] should be interpreted cautiously due to differences in exposure pathways, the detected levels remain relatively low.

The lead (Pb) levels detected in leaves (30.65 ± 1.76 ppm) and stems (24.85 ± 0.54 ppm) of *Y. decipiens* showed significant differences (*p* < 0.05) and were considerably higher than the limits considered safe for drinking water (10 μg L^−1^) and food products (0.5 mg kg^−1^) according to international standards [48,49]. These concentrations therefore indicate a potential concern for human consumption if plant material were ingested in significant amounts over extended periods. Although individual Pb isotopes (^206^Pb, ^207^Pb, ^208^Pb) were quantified by ICP-MS, these values represent isotopic signals of total lead and should not be interpreted as additive concentrations.

While most trace elements remained within international safety thresholds for plant tissues, Pb concentrations exceeded recommended limits for edible plant materials, warranting caution in potential food applications. However, direct comparisons between plant concentrations (ppm) and human intake limits (µg day^−1^) should be interpreted carefully, as bioavailability, processing effects, and actual consumption patterns were not evaluated in this study.

The differential accumulation observed between leaves and stems suggests organ-specific metal uptake or storage mechanisms. Such patterns may also reflect environmental conditions at the sampling site, including soil composition and metal bioavailability. Therefore, further studies assessing soil characteristics and plant–soil interactions are necessary to clarify whether these elevated concentrations result from environmental exposure or intrinsic accumulation capacity. Under controlled cultivation conditions and with appropriate monitoring of soil metal content, the biomass of *Y. decipiens* could still be explored for non-edible or industrial applications.

A different pattern was observed for arsenic (As). Concentrations in leaves (0.28 ppm) and stems (0.29 ppm) did not differ significantly (*p* > 0.05) and remained within internationally accepted safety values. The recommended safe intake for adults is 15 to 25 μg day^−1^, with a toxic limit of 3 mg day^−1^ after prolonged exposure [50], and the levels found in this plant are much lower than the reference values.

Soil contamination can significantly contribute to the accumulation of metals in plants [51], highlighting the importance of establishing rigorous controls in species used for food and medicinal purposes [52]. Although the presence of lead is a relevant finding, the levels detected in the leaves and stems of *Y. decipiens* do not necessarily imply a direct risk to the population, depending on consumption frequency and bioavailability, as this study corresponds to a basic characterization of the species rather than a comprehensive food safety assessment. Rather, these results suggest that *Y. decipiens* possesses a high metal absorption capacity, making it a potential candidate for phytoremediation processes in contaminated soils or water bodies with industrial (mining) or urban waste. However, several authors warn that the lack of quality control in plant products can allow for unusually high concentrations of contaminants, which could lead to cases of toxicity or poisoning [53]. Therefore, while *Y. decipiens* shows a mineral profile with essential trace elements and generally controlled toxicity, it is recommended to consider additional food safety and bioavailability studies to ensure safe use in human consumption or nutraceutical applications, since, for example, *Y. decipiens* contains metabolites with therapeutic applications. Accordingly, the detected concentrations should be interpreted within an environmental and physiological context, rather than as an immediate indication of food safety risk.

The results presented in Table 5 demonstrate the presence of metals and rare earth elements in the leaves and stems of *Y. decipiens*, with significant differences between organs. Although most elements were found within internationally accepted limits, Pb exceeded recommended thresholds, certain elements such as Tl, Bi, La, and Th lack clear reference values, highlighting the need for further food safety studies. These findings are relevant for assessing both the nutritional potential and toxicological risks, especially for vulnerable populations such as children and breastfeeding women.

Strontium (Sr), uranium (U), and vanadium (V) levels showed significant differences between leaves and stems (*p* < 0.05), although in all cases they remained within the safe limits established by international organizations [54]. For example, although strontium showed relatively high concentrations in leaves (0.32 ppm), these values did not exceed the reference toxicity index of 2 mg kg^−1^ day^−1^ [54], and the uranium (U) also did not exceed the reference limit reported in the literature (0.002 ppm) [54], suggesting a low toxicological risk. However, elements such as thallium (Tl), bismuth (Bi), lanthanum (La), and thorium (Th) lack clear reference values for their direct toxicological evaluation.

The concentrations of these elements in *Y. decipiens* were low and comparable to those reported in other edible species [55], although the differences observed between organs were also significant (*p* < 0.05). These results are consistent with previous studies that have documented the presence of metals and rare earth elements in common foods without establishing a clear risk relationship for humans [56,57,58]. The transfer of these elements to humans depends on factors such as the type of food, its origin, and preparation.

Finally, considering that *Y. decipiens* may have nutritional value, particularly considering its potential use as a plant-based nutritional resource, its elemental profile is relevant for food safety. Recent studies show that some processed products, such as infant formula, may contain potentially toxic trace elements [59], highlighting the importance of evaluating natural products like *Yucca* under similar criteria.

Table 6 shows the distribution of trace elements and rare earth elements in the leaves and stems of *Y. decipiens*, revealing significant differences (*p* < 0.05) between organs. Although most detected concentrations remained within ranges considered safe according to international standards, the observed differential accumulation reflects characteristic mineral translocation patterns of the species. These patterns may be relevant for both nutritional applications and for studies related to bioaccumulation and phytoremediation.

Other rare earth elements, such as holmium (Ho, 0.00548–0.00824 ppm) and erbium (Er, 0.0162–0.0240 ppm), also showed significant differences (*p* < 0.05) between plant organs, with a trend toward greater accumulation in leaves. However, these elements remained at low concentrations and below values reported as potentially toxic in previous studies [60,61]. The presence of gold (Au, 0.125–0.150 ppm) was consistent with the ranges reported in other edible plant species [55]. Overall, a significant differential accumulation (*p* < 0.05) was observed between leaves and stems, reflecting mineral translocation patterns characteristic of the species.

The relatively high concentrations of lead and certain rare earth elements in the leaves warrant particular attention. Despite the nutritional advantages of foliar tissues, the accumulation of these metals surpasses internationally accepted safety thresholds for edible plant materials. This suggests that the use of leaves for food or pharmaceutical purposes requires strict quality control, selective harvesting, or potential decontamination strategies such as chelation, adsorption-based purification, or controlled processing.

The organ-specific separation revealed by PCA reinforces the idea that *Y. decipiens* exhibits distinct mineral allocation strategies that reflect adaptive responses to semi-arid environments. The species’ ability to retain both essential and non-essential elements suggests potential multifunctional applications. While nutritionally promising, *Y. decipiens* may also warrant future evaluation for phytoremediation under controlled conditions in metal-contaminated soils. This duality underscores the importance of evaluating environmental context, tissue selection and final product safety before integrating *Y. decipiens* into food systems or bioactive ingredient pipelines.

Ultimately, the combined mineral, toxicological and PCA evidence supports a nuanced view: leaves provide superior nutritional potential but require stringent safety assessment, whereas stems, although less nutrient-dense, may serve as a phytotechnological asset for environmental applications. These insights highlight the need for organ specific valorization strategies to maximize the species’ biotechnological relevance while ensuring consumer safety. Overall, the PCA supports an organ-specific biomass valorization framework, reinforcing the need for selective use of leaves and stems according to nutritional, environmental, or biotechnological objectives under sustainable management schemes.

The multivariate patterns observed in the mineral composition of *Y. decipiens* reflect the combined influence of physiological compartmentalization, soil geochemistry, and the species’ inherent capacity for differential ion sequestration. Leaves consistently exhibited higher concentrations of essential macro and micronutrients, a pattern aligned with their metabolic demand for ions involved in photosynthesis, membrane stability, enzyme activation, and redox homeostasis. The dominance of K, Ca, Mg, P, Zn, Cu, and Mn in foliar tissues suggests that leaves function as nutritionally dense organs, potentially valuable for nutrient-enriched formulations or as raw material for functional ingredient extraction.

In contrast, stems showed elevated levels of mobile or redox-reactive toxic elements such as thallium, uranium isotopes and tungsten. This distribution implies a structural sequestration strategy, where the stem acts as a reservoir for contaminants translocated via the xylem. Although the concentrations detected remain below those typically associated with acute toxicity, their presence highlights the need for targeted organ selection when directing *Y. decipiens* towards industrial or nutraceutical applications.

The results illustrated in Figure 2a,b show that, after the first year, *Y. decipiens* reaches a more consistent growth phase. This pattern indicates that, once the initial establishment stage is overcome, the species exhibits relatively predictable growth, which could optimize harvest planning and biomass management in controlled production systems. The stabilization of height observed after 884 days suggests an upper limit on leaf development under the evaluated conditions, information relevant for the design of nursery cultivation programs and mass propagation strategies.

These findings are consistent with those reported by [62], who describe young individuals as having a rosette without an apparent stem, while mature individuals develop a single, unbranched trunk up to six meters tall, covered with dry leaves. This reinforces the characterization of *Y. decipiens* as a slow-growing species, especially during the early stages, and demonstrates that leaf accumulation and trunk formation require prolonged periods.

From an applied perspective, this pattern of slow and limited growth in the early years suggests that biomass production for nutritional or biotechnological purposes should prioritize the optimization of nurseries and controlled cultivation systems, considering harvest timing and the selection of plant organs according to their functional potential. Furthermore, information on the steady state growth phase can serve as a basis for subsequent studies on the extraction efficiency of bioactive compounds and the planning of agronomic trials focused on maximizing foliar yield.

Although the species shows relatively slow growth during early developmental stages, its capacity to produce mineral-rich foliar biomass under semiarid conditions highlights its potential as a sustainable plant resource for future biotechnological exploitation in arid and semiarid agroecosystems. Although the present study provides a detailed characterization of *Y. decipiens* biomass, the mineral and proximate composition of plant tissues may vary depending on species, soil characteristics, climate, and cultivation conditions. Therefore, the values reported here should be interpreted as representative of the population analyzed under the specific environmental conditions of the sampling site. Further studies evaluating *Y. decipiens* populations from different regions, as well as other species of the genus *Yucca*, will be necessary to determine the broader applicability of these findings. The characterization of underutilized native plant species such as *Y. decipiens* is essential to expand the portfolio of sustainable biomass resources adapted to semiarid ecosystems.

## 5. Conclusions

The integrated assessment of proximate composition, free amino acid content, multielemental profiling by ICP-MS, and growth performance revealed clear organ-specific differentiation in *Y. decipiens*. Leaves showed the highest dietary fiber content (58.93%) and elevated concentrations of essential macrominerals such as potassium, calcium, and magnesium. Stems, however, contained higher carbohydrate levels, protein content, and free amino acids, reflecting their structural and metabolic roles. Most detected elements remained within internationally accepted safety thresholds, although the presence of lead highlights the importance of monitoring environmental contamination and bioavailability. The observed organ-specific mineral partitioning supports selective biomass utilization strategies and highlights the potential of this species as an emerging crop for semiarid environments with possible applications in nutrition, agroindustry, and sustainable biotechnological systems.

## Figures and Tables

**Table 1:** Proximate composition of *Y. decipiens* leaves and stems. Values obtained from three independent replicates for each organ ± standard deviation. Different letters in the same row indicate a statistically significant difference (*p* < 0.05).

Variable (%)	Leaves (±SD)	Stems (±SD)
Total carbohydrates	12.83 ± 0.57 ^b^	28.83 ± 1.37 ^a^
Total sugars	9.63 ± 0.58 ^b^	23.37 ± 0.73 ^a^
Total fat	2.33 ± 0.46 ^a^	1.11 ± 0.68 ^b^
Ash	6.73 ± 0.11 ^b^	7.52 ± 0.19 ^a^
Protein	9.17 ± 0.40 ^a^	10.88 ± 0.48 ^b^
Moisture	6.09 ± 1.03 ^a^	5.78 ± 0.85 ^a^
Dietary fiber	58.93 ± 2.22 ^a^	40.10 ± 1.78 ^b^

**Table 2:** Mineral content in *Y. decipiens* leaves and stems. Values obtained from three independent replicates for each organ ± standard deviation. Different letters in the same row indicate a statistically significant difference (*p* < 0.05).

Mineral (ppm)	Leaves (±SD)	Stems (±SD)
Calcium	15,345.54 ± 1.63 ^a^	7628.34 ± 1.96 ^b^
Potassium	28,334.00 ± 7.76 ^a^	20,190.31 ± 3.90 ^b^
Magnesium	8155.24 ± 0.89 ^a^	2299.34 ± 6.32 ^b^
Sodium	252.81 ± 3.92 ^b^	304.68 ± 7.46 ^a^
Phosphorus	4845.25 ± 6.38 ^a^	3299.19 ± 4.20 ^b^

**Table 3:** Essential trace elements in *Y. decipiens* organs. Values represent the mean of three independent replicates for each organ ± standard deviation. Different letters in the same row indicate a statistically significant difference (*p* < 0.05).

Element (ppm)	Leaves (±SD)	Stems (±SD)
Cobalt	0.31 ± 0.01 ^b^	0.41 ± 0.04 ^a^
Chromium	25.41 ± 3.19 ^a^	21.95 ± 2.22 ^b^
Copper	256.71 ± 0.10 ^a^	160.51 ± 0.96 ^b^
Iron	587.46 ± 7.19 ^a^	1253.39 ± 9.71 ^b^
Manganese	53.90 ± 7.11 ^a^	23.82 ± 5.87 ^b^
Molybdenum	1.41 ± 0.08 ^a^	0.99 ± 0.02 ^b^
Selenium	0.06 ± 0.01 ^b^	0.05 ± 0.00 ^a^
Zinc	764.47 ± 2.89 ^a^	703.65 ± 2.62 ^b^

**Table 4:** Potentially toxic trace elements in organs of *Y. decipiens*. Values represent the mean of three independent replicates ± standard deviation. Different letters within the same row indicate statistically significant differences (*p* < 0.05).

Trace and Potentially Toxic Elements (ppm)	Leaves (±SD)	Stems (±SD)
Aluminum	464.13 ± 5.86 ^a^	483.20 ± 2.84 ^a^
Silver	0.02 ± 0.01 ^a^	0.01 ± 0.01 ^b^
Silicon	402.05 ± 8.66 ^a^	340.57 ± 3.19 ^b^
Arsenic	0.28 ± 0.01 ^b^	0.29 ± 0.01 ^a^
Barium	206.95 ± 0.12 ^a^	128.73 ± 2.87 ^b^
Beryllium	0.04 ± 0.00 ^a^	0.02 ± 0.00 ^b^
Cadmium	0.19 ± 0.01 ^b^	0.28 ± 0.01 ^a^
Cesium	0.16 ± 0.07 ^a^	0.15 ± 0.01 ^b^
Gallium	0.00 ± 0.00 ^a^	0.00 ± 0.00 ^b^
Mercury	0.01 ± 0.00 ^b^	0.01 ± 0.00 ^a^
Lithium	0.00 ± 0.00 ^b^	0.00 ± 0.00 ^a^
Nickel	0.02 ± 0.00 ^a^	0.02 ± 0.00 ^b^
Lead	30.65 ± 1.76 ^a^	24.85 ± 0.54 ^b^
Rubidium	0.01 ± 0.01 ^b^	0.01 ± 0.01 ^a^
Platinum	0.00 ± 0.00 ^a^	0.00 ± 0.00 ^a^
Antimony	0.18 ± 0.01 ^b^	0.28 ± 0.03 ^a^

**Table 5:** Rare-earth elements and selected trace metals in organs of *Y. decipiens*. Values represent the mean of three independent replicates ± standard deviation. Different letters within the same row indicate statistically significant differences (*p* < 0.05).

Trace and Potentially Toxic Elements (ppm)	Leaves (±SD)	Stems (±SD)
Tin	9.52 ± 1.32 ^b^	9.71 ± 1.62 ^a^
Strontium	0.32 ± 0.01 ^a^	0.24 ± 0.01 ^b^
Titanium	0.25 ± 0.01 ^a^	0.12 ± 0.01 ^b^
Thallium	0.03 ± 0.01 ^b^	0.03 ± 0.01 ^a^
Uranium	0.07 ± 0.01 ^b^	0.09 ± 0.01 ^a^
Vanadium	0.00 ± 0.00 ^a^	0.00 ± 0.00 ^b^
Bismuth	7.96 ± 0.17 ^b^	8.42 ± 0.22 ^a^
Thorium	0.02 ± 0.00 ^a^	0.02 ± 0.00 ^b^
Scandium	0.00 ± 0.00 ^a^	0.00 ± 0.00 ^b^
Germanium	0.00 ± 0.00 ^a^	0.00 ± 0.00 ^b^
Yttrium	0.00 ± 0.00 ^a^	0.00 ± 0.00 ^b^
Zirconium	0.00 ± 0.00 ^b^	0.00 ± 0.00 ^b^
Niobium	0.00 ± 0.00 ^a^	0.00 ± 0.00 ^b^
Rhodium	0.00 ± 0.00 ^a^	0.00 ± 0.00 ^a^
Palladium	0.00 ± 0.00 ^a^	0.00 ± 0.00 ^a^
Lanthanum	0.01 ± 0.00 ^a^	0.01 ± 0.00 ^a^
Cerium	0.55 ± 0.01 ^a^	0.32 ± 0.01 ^b^
Praseodymium	0.07 ± 0.01 ^a^	0.04 ± 0.01 ^b^
Neodymium	0.26 ± 0.01 ^a^	0.15 ± 0.01 ^b^
Samarium	0.05 ± 0.01 ^a^	0.03 ± 0.01 ^b^
Europium	0.01 ± 0.00 ^a^	0.01 ± 0.00 ^b^
Gadolinium	0.05 ± 0.01 ^a^	0.03 ± 0.01 ^b^
Terbium	0.01 ± 0.00 ^a^	0.00 ± 0.00 ^b^

**Table 6:** Additional trace metals with organ-specific accumulation in organs of *Y. decipiens*. Values obtained from three independent replicates for each organ ± standard deviation. Different letters in the same row indicate a statistically significant difference (*p* < 0.05).

Trace and Potentially Toxic Elements (ppm)	Leaves (±SD)	Stems (±SD)
Lanthanum	12.03 ± 1.59 ^b^	12.04 ± 1.01 ^a^
Dysprosium	0.04 ± 0.01 ^a^	0.03 ± 0.01 ^b^
Holmium	0.01 ± 0.00 ^a^	0.01 ± 0.00 ^b^
Erbium	0.02 ± 0.01 ^a^	0.02 ± 0.01 ^b^
Thulium	0.00 ± 0.00 ^a^	0.00 ± 0.00 ^b^
Ytterbium	0.02 ± 0.01 ^a^	0.01 ± 0.00 ^b^
Lutetium	0.00 ± 0.00 ^a^	0.00 ± 0.00 ^b^
Hafnium	0.02 ± 0.01 ^a^	0.02 ± 0.05 ^b^
Tantalum	0.07 ± 0.01 ^a^	0.06 ± 0.01 ^b^
Tungsten	0.27 ± 0.02 ^b^	0.35 ± 0.01 ^a^
Rhenium	0.00 ± 0.00 ^a^	0.00 ± 0.00 ^b^
Gold	0.13 ± 0.02 ^b^	0.15 ± 0.01 ^a^

**Table 7:** Cumulative explanatory variance of six principal components for the mineral composition of *Y. decipiens* leaves and stems.

Principal Component	Eigenvalue	Proportion of Variance	Cumulative Variance
PC1	51.77	0.70	0.70
PC2	6.06	0.08	0.78
PC3	3.86	0.05	0.83
PC4	2.55	0.03	0.87
PC5	2.24	0.03	0.90
PC6	1.86	0.03	0.92

**Table 8:** Loadings of principal components for the mineral composition of *Y. decipiens* leaves and stems.

Mineral	PC1	PC2	PC3	PC4	PC5
LI	0.14	−0.08	0.00	−0.06	−0.05
BE	−0.13	0.07	0.01	0.10	0.03
NA	0.14	−0.04	0.02	0.03	0.02
MG	−0.14	0.04	0.00	−0.01	−0.01
AL	0.06	−0.04	0.37	−0.25	−0.11
SI	−0.09	0.27	−0.02	0.12	−0.03
P	−0.14	0.02	0.00	−0.02	−0.05
K	−0.14	0.02	−0.01	0.00	−0.02
CA	−0.14	0.03	0.00	0.00	−0.01
SC	−0.14	−0.02	−0.03	−0.03	−0.04
TI	−0.14	0.01	−0.02	−0.02	−0.05
V	−0.14	0.00	0.01	0.06	−0.11
CR	−0.10	−0.16	0.12	−0.19	0.20
MN	−0.14	0.04	−0.01	−0.01	0.01
FE	0.14	−0.04	0.01	−0.02	−0.01
CO	0.13	−0.01	0.00	0.05	−0.02
NI	−0.11	−0.17	0.11	−0.17	0.12
CU	−0.14	0.02	−0.03	−0.05	0.08
ZN	−0.06	0.27	0.27	−0.07	0.09
GA	−0.13	−0.14	0.06	0.03	−0.20
GE	−0.11	−0.24	−0.08	−0.09	−0.01
AS	0.03	0.07	−0.39	−0.26	−0.28
SE	−0.12	−0.14	−0.06	−0.19	0.10
RB	0.12	−0.16	−0.03	0.01	−0.09
SR	−0.14	0.01	−0.02	−0.01	−0.05
Y	−0.14	−0.09	−0.01	0.04	−0.02
ZR	0.04	0.04	0.34	−0.07	0.21
NB	−0.14	−0.03	−0.02	−0.02	−0.03
MO	−0.14	0.03	0.01	0.01	−0.01
RH	−0.05	0.21	−0.10	−0.37	−0.05
PD	−0.14	0.02	−0.02	0.00	−0.02
AG	−0.14	−0.02	0.06	−0.01	−0.05
CD	0.14	−0.03	−0.01	0.00	0.00
SN	0.06	0.24	−0.06	0.08	−0.13
SB	0.12	−0.09	0.15	−0.11	0.00
CS	−0.09	−0.10	−0.22	−0.15	−0.07
BA	−0.14	0.03	−0.01	−0.03	−0.01
LA	−0.14	0.01	0.03	0.04	−0.11
CE	−0.14	0.02	−0.01	0.00	−0.01
PR	−0.14	−0.08	−0.02	0.01	0.03
ND	−0.14	0.03	0.04	0.04	0.02
SM	−0.13	−0.14	0.00	0.02	0.01
EU	−0.14	−0.06	−0.07	−0.09	−0.03
GD	−0.14	−0.07	0.04	−0.05	−0.01
TB	−0.13	−0.08	0.13	0.02	−0.01
DY	−0.13	−0.11	0.09	0.06	0.11
HO	−0.14	−0.04	0.05	−0.04	−0.01
ER	−0.14	0.01	−0.03	−0.03	0.02
TM	−0.13	−0.14	0.07	0.04	0.07
YB	−0.13	0.02	0.09	−0.08	−0.08
LU	−0.12	−0.14	−0.02	−0.05	−0.08
HF	−0.12	−0.10	−0.04	−0.15	−0.11
TA	−0.08	0.03	0.00	−0.02	0.12
W	0.13	−0.13	0.05	0.02	−0.01
RE	−0.14	0.02	0.01	0.00	0.01
PT	0.00	−0.07	−0.18	−0.14	0.50
AU	0.13	−0.13	0.04	0.00	−0.09
HG	0.08	0.24	0.05	−0.25	0.00
TL	0.00	−0.32	−0.15	−0.06	−0.20
PB	−0.10	0.24	−0.16	0.09	0.02
BI	0.11	0.07	0.12	−0.26	−0.11
TH	−0.03	−0.10	0.22	0.15	−0.48
U	0.13	−0.02	−0.17	−0.07	0.01

Note: Element codes correspond to the elements quantified by ICP-MS.

## Data Availability

The datasets generated and analyzed during the current study are available from the corresponding author on reasonable request.
